# Influence of Sociodemographic Factors on Level Stress and Coping Strategies of Nurses and Midwives Caring for Newborns with Lethal Defects

**DOI:** 10.3390/nursrep15040116

**Published:** 2025-03-21

**Authors:** Katarzyna Anna Urbańska, Beata Naworska, Agnieszka Drosdzol-Cop

**Affiliations:** 1 Neonatology Unit, BCM The Guardian Angels Hospital of the Brothers Hospitallers of St. John of God in Katowice, Markiefki 87, 40-211 Katowice, Poland; 2 Department of Gynaecology and Obstetrics, Faculty of Health Sciences in Katowice, Medical University of Silesia in Katowice, 40-751 Katowice, Poland; bnaworska@sum.edu.pl; 3 Chair and Department of Gynecology, Obstetrics and Oncological Gynecology, Faculty of Medical Sciences in Katowice, Medical University of Silesia in Katowice, 40-211 Katowice, Poland; adrosdzol@sum.edu.pl

**Keywords:** level stress, sociodemographic factors, coping strategies, nurses, midwives, lethal defects, palliative care, neonatal nursing, congenital abnormalities

## Abstract

**Introduction:** Nurses and midwives caring for newborns with lethal defects experience significant emotional stress. Understanding coping strategies and the factors influencing stress is crucial for improving their well-being and ensuring high-quality care. **Objectives:** The aim of this study was to identify the coping strategies used by nurses and midwives in stressful situations and to analyse the relationship between stress levels and selected sociodemographic and professional factors. **Methods:** A cross-sectional study was conducted in the second quarter of 2023 among 307 nurses and midwives working in neonatal and obstetric wards in the Silesian metropolitan area, Poland. A diagnostic survey method was applied using a standardised questionnaire. The Perceived Stress Scale (PSS-10) and the MINI-COPE Inventory were used to assess stress levels and coping mechanisms. A stratified random sampling method was employed to ensure representation from various professional backgrounds. Data analysis was conducted using descriptive statistics, chi-square tests, Spearman’s correlation, and Cohen’s d coefficient, with statistical significance set at *p* < 0.05. **Results:** High stress levels were associated with shorter professional experience, frequent exposure to lethal defects, and emotional discomfort in interactions with grieving families. The most commonly used coping strategies were active coping (M = 2.06, SD = 0.635) and planning (M = 1.95, SD = 0.590), whereas self-blame (M = 1.20, SD = 0.714, *p* < 0.001) and denial (M = 0.88, SD = 0.751, *p* < 0.001) were linked to higher stress levels. Positive reinterpretation (r = −0.211, *p* < 0.001) and seeking emotional support (r = −0.129, *p* = 0.024) correlated with lower stress levels. Nurses and midwives with secondary education reported higher stress levels compared to those with higher education (χ^2^(10) = 30.651, *p* = 0.001). Work experience played a role, with moderate stress levels most frequently observed among those with 2–5 years of professional experience (χ^2^(14) = 24.023, *p* = 0.046). Emotional involvement, particularly supporting parents during their farewell to the child (69.1%), was identified as the most stressful aspect of their work. **Conclusions:** Promoting adaptive coping strategies, such as positive reinterpretation and emotional support, can help reduce stress and improve the well-being of nurses and midwives. Implementing psychological support programmes and stress management training is essential for maintaining high-quality neonatal care.

## 1. Introduction

Nursing is an extremely demanding and stressful profession that affects both the physical and mental health of nurses and midwives. The sources of stress are emotionally challenging situations, such as caring for critically ill patients or the need to manage the emotions of patients and their families [1]. This profession is associated with high levels of emotional, psychological, and physical strain, often leading to dissatisfaction with work [2]. More than 50% of nurses experience high levels of stress, making their profession more demanding than other medical fields, due to their direct involvement in patient care [1,3].

While a key aspect of midwifery is supporting patients through natural processes such as pregnancy and childbirth, in practice, they often face difficult and stressful experiences. These include complications during pregnancy, the birth of a child with serious illness or a lethal defect, miscarriage or stillbirth, all of which can lead to significant psychological distress among medical staff [4]. Death and dying are among the most challenging aspects of nursing and midwifery work, triggering extreme emotions and stress-related symptoms [1].

Studies show that occupational stress negatively affects the health of nurses and midwives, increasing the risk of health problems, absenteeism, errors, and burnout [2,5,6]. The death of a child is a traumatic experience not only for the mother and her family but also for the nursing staff, which can reduce the quality of care provided [7].

The term “stress” is widely used in various scientific fields and everyday language, yet there is no universally accepted definition. According to Alhabri [8], stress is a state of discomfort resulting from situations perceived as excessively demanding or beyond an individual’s coping abilities. Goudarzian et al. highlight that nurses encounter various types of stress, including occupational, environmental, psychological, and emotional stress [1]. Occupational stress can result from heavy workloads, excessive empathy for patients’ suffering, or a sense of responsibility [9]. The National Labour Inspectorate defines occupational stress as a state in which employees experience psychological discomfort due to work conditions or demands exceeding their abilities [10].

Additional factors triggering stress for nurses and midwives include delivering distressing news to patients and their families, life-threatening situations, ethical dilemmas, and the need to provide end-of-life care information [11,12,13,14]. Long-term exposure to a stressful work environment not only affects staff health but also increases the need for stress coping strategies [15]. A strategic approach includes both physical and psychological actions aimed at mitigating the negative effects of stress [16].

In recent years, healthcare organisations have implemented various interventions to support staff in managing stress. Growing interest is being given to stress management methods and improving the mental health of nurses, such as mindfulness, meditation, psychological support, and relaxation techniques [17,18]. While some of these interventions show promising results, studies by Chesak et al. [19] and Li et al. [20] indicate their moderate effectiveness, emphasising the need for further research. According to De Diego-Cordero et al. [21], nurses use a wide range of stress-coping strategies, with one of the key methods being reliance on religious and spiritual beliefs.

The phenomenon of stress in nursing includes reactions that arise in response to demands and pressures exceeding an individual’s abilities [22].

In Poland, a three-tier referral system of care for pregnant women and newborns operates, providing differentiated levels of care depending on the complexity of the pregnancy and the health status of the newborn [23,24]:

1. Level I—provides basic care for women with physiological pregnancies and healthy newborns. At this level, prenatal education, childbirth preparation, and breastfeeding and childcare education are conducted. Care focuses on prevention and early detection of abnormalities, which is crucial in reducing the risk of perinatal complications.
2. Level II—pertains to care for pregnancies at moderate risk and treatment of newborns with mild health problems. Level II hospitals have neonatology departments that care for premature infants and those needing medical supervision but not intensive therapy. Nurses and midwives at this level often face difficult clinical decisions and provide support to families dealing with complications during pregnancy and childbirth.
3. Level III—provides highly specialised care for high-risk pregnancies and intensive therapy for newborns born with serious defects and illnesses requiring advanced treatment. At this level, neonatal intensive care units (NICUs) are equipped with the latest medical technologies, such as mechanical ventilation, ECMO therapy, and advanced surgical procedures. Nurses working at this level must demonstrate high mental resilience and expertise in caring for critically ill patients.

Each of these levels involves varying degrees of psychological and physical strain for the medical staff. Working in Level II and III departments often requires coping with emotionally challenging situations such as the birth of a child with a fatal defect, prolonged hospitalisation of a newborn, or supporting families through the grieving process.

Therefore, implementing strategies to reduce stress among nursing staff and providing appropriate psychological support is of crucial importance.

Nursing staff working in neonatal intensive care units (NICUs) make every effort to provide the best care for critically ill children and their families. The death of a newborn and the grieving process significantly impact the staff, often causing deep emotional stress for nurses and midwives. Working in a NICU, Maternity Ward, Delivery Room, neonatology department, or perinatal hospice involves constant contact with suffering newborns and their families, leading to serious consequences for the mental, emotional, and physical health of the staff. Prolonged exposure to difficult situations, such as the death of patients, helplessness in the face of newborn suffering, or the need to support grieving families, can lead to post-traumatic stress disorder (PTSD) [25], burnout, and depression. Unfortunately, nurses and midwives do not always receive appropriate support from family, colleagues, or employers, making this issue an important topic for further research. The introduction of psychological support programmes, stress management training, and regular supervision could help improve the mental health of staff and the quality of care provided to patients.

## 2. Objectives

1. Identifying coping strategies in stressful situations. The first aim of the study was to identify specific coping strategies used by nurses and midwives in the face of stressful situations while caring for newborns with lethal malformations. This involved examining the coping mechanisms, resilience and potential adaptive or maladaptive behaviours that healthcare workers adopt to cope with the emotional and professional challenges associated with such critical care scenarios.

2. Exploring the relationship between stress levels and socio-demographic/professional factors. The second aim was to determine how stress levels among nurses and midwives correlated with selected socio-demographic and professional factors. This involved examining variables such as age, years of experience, work environment, educational level and personal background to assess their impact on the perception and management of stress in the context of palliative neonatal care.

## 3. Material and Methods

The study was conducted in the second quarter of 2023 in medical centres with secondary and tertiary levels of reference within the Silesian agglomeration in Poland. In order to obtain an opinion on the necessity of ethical approval for the study, a request was submitted to the Bioethics Committee of the Medical University of Silesia in Katowice. According to the decision of the Bioethics Committee of the Medical University of Silesia in Katowice (approval number: PCN/CBN/0052/KB/33/23 dated 28 March 2023), the study was not classified as a medical experiment and did not require ethical review by the Committee. Participation in the study was voluntary, and respondent anonymity was ensured. 

A request for permission to conduct the study was sent to seven hospitals with tertiary-level reference in the three-tier system of care for pregnant women and neonates operating in the Silesian Voivodeship. Due to the refusal of three tertiary-level hospitals, an additional secondary-level hospital, which includes a perinatal hospice, was included in the study. In the Silesian Voivodeship, tertiary-level reference hospitals comprise University Hospital Clinics and Provincial Specialist Hospitals, secondary-level reference hospitals include Provincial Hospitals, Municipal Hospitals, and one private hospital, while primary-level reference hospitals include District Hospitals and other private hospitals. According to the report of the Supreme Council of Nurses and Midwives in Poland [26], published in 2023, there were 39,685 registered nurses and 4905 registered midwives (a total of 44,950 nurses and midwives) in the Silesian Voivodeship.

Initially, the maximum margin of estimation error was assumed to be 5%, which resulted in a required sample size of 381 participants. However, due to the nature of the study and the target group—nurses and midwives performing their duties in centres within the level III reference system of the three-tiered care system for pregnant women and newborns—the representative sample ultimately consisted of 306 individuals.

The study was quantitative in nature, and the study group comprised nurses and midwives working in neonatal, intensive care, and neonatal pathology units, obstetric wards, delivery suites, and perinatal hospices. The questionnaire was distributed to 340 respondents, with 316 returned, of which 307 complete questionnaires were qualified for analysis.

The sample size was calculated using the formula for a finite population:

$$n=\frac{0.5\left(1-P\right)}{\frac{e^{2}}{Z^{2}}+\frac{P\left(1-P\right)}{N}}=\frac{0.5\left(1-0.5\right)}{\frac{0.05^{2}}{1.96^{2}}+\frac{0.5\left(1-0.5\right)}{44950}}=380.87\;n=\frac{0.5\left(1-P\right)}{\frac{e^{2}}{Z^{2}}+\frac{P\left(1-P\right)}{N}}=\frac{0.5\left(1-0.5\right)}{\frac{0.05^{2}}{1.96^{2}}+\frac{0.5\left(1-0.5\right)}{1503}}=305.95$$

where *N*—population size, *Z*—value derived from the assumed confidence level (for 95%, *Z* = 1.96), *P*—estimated proportion in the population, *e*—acceptable estimation error.

A diagnostic survey method was applied, using a proprietary questionnaire that included questions regarding sociodemographic and occupational factors such as education, workplace, professional experience, and reasons for choosing the current workplace. Additionally, it contained questions on the frequency of care provided to patients diagnosed prenatally with a lethal foetal anomaly, exposure to neonatal death, nursing procedures related to the care of neonates with lethal anomalies, the degree of discomfort experienced, and factors contributing to psychological burden. The questionnaire was supplemented with standardised research tools: the Mini-COPE Inventory for measuring coping strategies and the Perceived Stress Scale (PSS-10).

To measure coping strategies, the shortened version of the COPE questionnaire by Carver, Scheier, and Weintraub, adapted into Polish by Juczyński and Ogińska-Bulik [27], was used. The Polish version of Mini-COPE consists of 28 statements assigned to 14 coping strategies. Respondents rated their behaviours on a four-point Likert scale (0—almost never, 1—rarely, 2—often, 3—almost always). 

The Perceived Stress Scale (PSS-10) by Cohen, Kamarck, and Mermelstein, adapted into Polish by Juczyński and Ogińska-Bulik [28], comprises 10 questions assessing the intensity of stress related to life situations. Respondents rated the frequency of specific feelings on a scale from 0 (never) to 4 (very often). The total score ranged from 0 to 40 points, with values of 0–13 indicating low stress, 14–26 moderate stress, and 27 or more high stress levels. 

The psychometric properties of the standardised tools used are presented below.

Mini-COPE:

• Internal consistency (reliability): Guttman’s coefficient is 0.87, indicating high reliability of the tool.
• Score range: 0–3 for each strategy.
• Score distributions: Problem-focused coping strategies, such as active coping (M = 2.06, SD = 0.635) and planning (M = 1.95, SD = 0.590), have the highest mean values.
○ Less adaptive strategies, such as substance use (M = 0.66, SD = 0.712) and denial (M = 0.88, SD = 0.751), show lower means.
○ Skewness and kurtosis indicate slight deviations from a normal distribution.
• PSS-10 (Perceived Stress Scale):
• Internal consistency (reliability): Cronbach’s alpha is 0.86, suggesting high reliability of the tool.
• Score range: 0–4 per question, 0–40 for the entire scale.
• Score distributions: The highest mean scores were observed for questions related to feeling nervous and tense (M = 2.70, SD = 0.883) and perceived ability to handle problems (M = 2.67, SD = 0.831). The lowest values were recorded for feeling helpless in the face of increasing difficulties (M = 1.85, SD = 0.914), which may suggest that respondents experienced extreme negative emotional states less frequently.

Statistical analysis was conducted using IBM SPSS 25.01 and Microsoft Excel. The following statistical methods were applied:

- Descriptive statistical analysis (measures of central tendency and dispersion) to characterise quantitative variables.
- Frequency analysis—percentage distributions of qualitative variables.
- Chi-square tests and contingency tables to assess relationships between nominal and ordinal variables.
- Cohen’s d effect size measure to determine the magnitude of differences between groups.
- Spearman’s correlation coefficient for quantitative variables deviating from normal distribution.

A significance level of α = 0.05 was adopted (results with *p* < 0.05 were considered statistically significant), with a confidence level of 95%.

## 4. Results

Description of the Cross-Tabulation of PSS10 Scores with Various Demographic and Professional Variables

The cross-tabulation analysis examined the relationship between PSS10 (Perceived Stress Scale) scores and various demographic and professional variables.

The analysis revealed a significant association between education level and categorical distribution (χ^2^(10) = 30.651, *p* = 0.001, Cohen’s *d* = 0.2234). Individuals with a secondary medical high school education (66.7%) and a bachelor’s degree (56.3%) were most frequently classified in the high category, suggesting that these educational backgrounds may provide sufficient preparation for excelling in their professional roles. 

Interestingly, individuals with a post-secondary vocational school education were predominantly in the medium category (83.3%), indicating that this level of education might offer a solid but not necessarily advanced foundation for professional performance. Notably, all participants with a doctoral degree fell into the medium category (100.0%), which may suggest that holding a doctorate does not directly translate to superior performance or may reflect that individuals in these roles undertake different responsibilities that do not align with the assessed criteria.

Higher levels of education generally correlate with a greater likelihood of classification in the high category. However, doctoral-level education does not appear to confer a clear advantage in this specific professional context.

A statistically significant relationship was observed between years of service and category distribution (χ^2^(14) = 24.023, *p* = 0.046, Cohen’s *d* = 0.1978). The 11–15 years (53.7%) and 16–20 years (51.2%) groups had the highest proportion of individuals in the high category, indicating that experience contributes positively to professional performance.

Employees with the shortest tenure (up to one year) were predominantly in the medium category (63.6%), which is expected as they are still acquiring experience and adapting to their roles. In contrast, those with 21–25 years of service exhibited a more even distribution across all categories, suggesting that beyond a certain threshold, experience alone may not be a definitive factor in high performance.

Professional experience is positively associated with higher performance, particularly after six years in the department. However, after 20–25 years, the impact of additional experience appears to plateau, possibly due to factors such as routine, job fatigue, or changes in work dynamics.

No statistically significant relationship was found between workplace setting and category distribution (χ^2^(8) = 7.984, *p* = 0.435, Cohen’s *d* = 0.1140). Nevertheless, some differences were observed. The highest proportions in the high category were found in the neonatal intensive care unit (47.1%), Maternity Ward (48.0%), and Delivery Block (46.3%), suggesting that these environments may offer greater opportunities for skill development or require higher levels of professional engagement.

Conversely, the perinatal hospice showed an unusual trend, with an even distribution between the low (40.0%) and high (40.0%) categories. This may indicate that individuals in this setting either thrive due to personal dedication or struggle due to the unique emotional and psychological challenges of the role.

While no direct correlation was found between workplace setting and performance, the nature of the department may influence individual development. Units with higher patient turnover and acute care demands (e.g., NICU) seem to foster higher performance levels.

A significant association was found between the reason for workplace choice and categorical distribution (χ^2^(10) = 33.477, *p* < 0.001, Cohen’s *d* = 0.2335). The highest proportions in the high category were observed among individuals who chose their workplace based on the decision of superiors (83.3%)**,** a sense of calling (55.0%), and a desire to help (51.7%). This suggests that intrinsic motivation and external expectations play a crucial role in professional effectiveness.

Conversely, those who selected their workplace due to financial conditions were predominantly in the medium category (54.5%), implying that while financial incentives may sustain motivation, they are not necessarily linked to high performance. 

Employees driven by intrinsic motivation (such as a sense of calling) or external professional guidance (such as superior recommendations) are more likely to achieve high levels of performance. In contrast, financial motives alone do not appear to foster excellence. The details are in Table 1.

The study analysed various aspects of caregivers’ work in the context of caring for newborns with prenatally diagnosed lethal conditions. The focus was on the frequency of contact with such newborns and the factors that may influence the psychological burden on caregivers, such as discomfort, stress, and emotional involvement. The statistical analyses (chi-square tests, χ^2^) and effect size calculations using Cohen’s d coefficient allowed for the drawing of significant conclusions regarding these relationships.

Frequency of Patients with Prenatally Diagnosed Lethal Conditions. The analysis revealed a significant difference in the distribution of responses to the question regarding the frequency of encounters with patients with prenatally diagnosed lethal conditions (χ^2^(6) = 35.123, *p* < 0.001). The majority of respondents (55.6%) encountered such patients “several times a year”, and 44.4% reported care “once a quarter”. The responses in the “several times a month” category amounted to 54.1%.

The high percentage of caregivers indicating frequent encounters with patients in cases of prenatal lethal diagnoses suggests that this is a frequent aspect of work in neonatal care. It is worth noting that the frequency of these cases correlates with higher emotional burdens, which may influence how staff cope with such challenging situations.

Cohen’s d = 0.2392 indicates a small to medium effect, meaning that the frequency of encountering patients with lethal conditions has a moderate impact on caregivers’ emotional and professional responses.

Frequency of Contact with Dying Newborns. A significant difference in the frequency of contact with dying newborns was evident in the analysis (χ^2^(6) = 30.496, *p* < 0.001). The most frequent responses were in the “several times a year” category (45.7%), followed by “several times a month” (65.5%). In the “once a quarter” category, responses from 57.1% of respondents were recorded.

Close contact with dying newborns is one of the most difficult experiences for a neonatologist. The frequency of such cases affects the emotional condition of caregivers, who must deal with challenging, traumatic experiences, leading to increased stress.

Cohen’s d = 0.2229 indicates a moderate effect, suggesting that the frequency of contact with dying newborns significantly influences caregivers’ emotions.

Frequency of Performing Nursing Tasks Related to the Care of a Newborn with a Lethal Condition. In the studied group, the most common responses were regarding performing nursing tasks “several times a month” (61.8%), followed by “several times a year” (46.7%) and “once a quarter” (48.1%). The chi-square test showed statistical significance (χ^2^(6) = 31.510, *p* < 0.001).

More frequent nursing tasks are associated with higher levels of responsibility and greater stress. Caregivers who more often care for newborns with lethal conditions face both technical and emotional challenges, which may cause increased discomfort and difficulty.

Cohen’s d = 0.2265 suggests that the frequency of performing such tasks has a moderate impact on caregivers’ stress and emotional exhaustion.

Degree of Discomfort Experienced in the Care of a Newborn with a Lethal Condition. The analysis showed that the degree of discomfort depends on the frequency of contact with newborns with lethal conditions. The majority (74.4%) experienced “very large” discomfort, particularly with frequent interactions. In the “small/minimal” and “medium” discomfort categories, people with frequent contact were clearly dominant.

The experience of working with dying children and newborns with lethal conditions is associated with significant stress and emotional burden. The more frequent the contact, the higher the level of discomfort. This indicates the need for psychological support for caregivers who regularly face such traumatic situations.

Cohen’s d = 0.2212 indicates a small to medium effect, suggesting that more frequent interactions are associated with greater discomfort.

Factors Affecting Psychological Burden Considered to Cause Stress in the Care of a Newborn with a Lethal Condition.

Time Pressure: A significant relationship was observed (χ^2^(2) = 7.326, *p* = 0.026). The majority of respondents (69.6%) considered time pressure as a major stressor. Cohen’s d = 0.1092 indicates a small effect. Close Contact with Death: A significant difference was noted (χ^2^(2) = 13.610, *p* = 0.001), where 76.2% of respondents identified death as a stressful factor. Cohen’s d = 0.1489 indicates a medium effect. Helplessness or Limited Ability to Help: This factor also contributed to caregivers’ stress (χ^2^(2) = 6.903, *p* = 0.032). Around 43.9% of respondents felt helpless in the face of newborns’ lethal conditions. Cohen’s d = 0.1060 indicates a small effect. Excessive Emotional Involvement: No significant difference was found (χ^2^(2) = 3.205, *p* = 0.201), with a minimal effect (Cohen’s d = 0.0722). Close Contact with Parents’ Suffering: No significant differences were observed (χ^2^(2) = 2.309, *p* = 0.315), with a minimal effect (Cohen’s d = 0.0613). Close Contact with the Child’s Suffering: A result close to significance was observed (χ^2^(2) = 5.579, *p* = 0.061), with a small effect (Cohen’s d = 0.0953).

The analysis indicates that frequent exposure to caring for newborns with lethal conditions is associated with higher levels of discomfort and stress among caregivers. Factors such as time pressure, proximity to death, and helplessness in difficult situations are significant sources of stress. However, some factors, such as excessive emotional involvement or contact with the suffering of parents, did not have a large impact on stress levels. The results suggest the need for psychological and educational support for neonatal care workers to help them better cope with the difficult experiences associated with caring for dying children. The details are in Table 2.

The descriptive statistics from the Mini-COPE questionnaire reveal a wide range of coping strategies used by the participants in response to challenging situations.

The analysis of basic descriptive statistics for the Mini-COPE tool indicates a variation in stress-coping strategies within the studied group (N = 307). Below, the results for individual variables are discussed, taking into account their mean values (M), standard deviation (SD), median (Me), and range of scores, as well as skewness and kurtosis indices.

Most Frequently Used Strategies

Problem-focused coping strategies, such as active coping (M = 2.06, SD = 0.635) and planning (M = 1.95, SD = 0.590), obtained the highest mean values. This suggests that the respondents frequently engage in actions aimed at constructively solving problems. The negative skewness values for these strategies (−0.91 and −0.64) indicate an asymmetrical distribution with a predominance of higher values, confirming their dominance in the studied group.

Emotion-Focused Strategies

Emotion regulation strategies, such as positive reframing (M = 1.81, SD = 0.690) and acceptance (M = 1.86, SD = 0.574), also achieved relatively high scores. Their slight skewness (−0.54 and −0.34) and kurtosis values indicate a distribution close to normal.

Seeking emotional support (M = 1.79, SD = 0.716) and seeking instrumental support (M = 1.76, SD = 0.663) were also relatively frequently used, suggesting that respondents significantly rely on social support in difficult situations.

Less Adaptive Strategies

Less adaptive strategies, such as denial (M = 0.88, SD = 0.751) and substance use (M = 0.66, SD = 0.712), had lower mean values. Their positive skewness (ranging from 0.50 to 0.69) indicates a distribution shift towards lower scores, meaning that most participants rarely use these mechanisms.

Self-blame (M = 1.20, SD = 0.714) and behavioural disengagement (M = 0.95, SD = 0.689) also obtained relatively low scores, suggesting that these strategies are not predominantly employed in the studied group.

Analysis of Score Distributions

Most variables show skewness within the range of −1 to 1, indicating moderate asymmetries in distribution. The kurtosis values fluctuate around 0, suggesting slight deviations from normality. An exception is active coping, where kurtosis is 1.53, meaning that this score is more concentrated around the mean compared to a normal distribution.

Dominance of Adaptive Strategies

The studied group more frequently applies adaptive strategies, such as active coping, planning, and positive reframing. This indicates a constructive approach to problems and an ability to regulate emotions effectively.

Rare Use of Avoidant and Maladaptive Strategies

Strategies based on avoiding problems (e.g., denial, substance use) are used significantly less frequently, suggesting that the respondents do not rely heavily on escapist mechanisms when dealing with stress.

Importance of Social Support

Relatively high scores for emotional and instrumental support-seeking strategies highlight the importance of interpersonal relationships in the stress-coping process. This suggests a preference for strategies that engage social resources.

Potential Implications for Psychological Interventions

The results indicate the need to promote adaptive coping strategies and minimise maladaptive ones through appropriate psychological interventions, such as stress management training and psychological support programmes.

In summary, the psychometric analysis of the Mini-COPE tool indicates a predominant use of constructive stress-coping strategies within the studied group, which may have positive consequences for the participants’ psychological well-being. Details can be seen in Table 3.

The Spearman correlation analysis between the PSS-10 score and coping strategies from the Mini-COPE questionnaire reveals significant relationships between stress levels and coping methods.

Positive correlations, indicating higher stress levels, were found with strategies like self-blame, giving up, denial, turning to religion, and substance use. The strongest positive correlation was with self-blame, suggesting that individuals who frequently use this strategy tend to experience higher stress.

On the other hand, negative correlations, indicating lower stress levels, were found with positive reinterpretation and seeking emotional support. These strategies may be linked to more adaptive coping, contributing to lower stress.

Other strategies, such as active coping, planning, acceptance, sense of humour, seeking instrumental support, and distraction, showed no significant correlations with stress levels, indicating that their relationship with stress was not confirmed in this sample.

In conclusion, the analysis suggests that strategies like positive reinterpretation and seeking emotional support may help reduce stress, while strategies such as self-blame, denial, and giving up are associated with higher stress levels. These findings highlight the importance of promoting more adaptive coping strategies for stress management (Table 4).

What is the Most Stressful Aspect of Caring for a Newborn with a Lethal Defect?

Table 5 illustrates the percentage of responses regarding the most stressful actions for medical staff caring for newborns with lethal defects.

The majority of respondents (69.1%) identified accompanying parents during their farewell to the child as the most stressful task. This emotionally intense interaction involves confronting the newborn’s death and the parents’ profound grief.

The second most commonly mentioned stressor (19.2%) was preparing keepsakes, such as footprint impressions. Despite its technical nature, the symbolic significance of this task can be emotionally taxing.

Providing palliative care without life-prolonging treatment and handling funeral documentation were cited as stressful by 13% of respondents. These activities require both medical expertise and emotional sensitivity when supporting grieving families.

Post-mortem care, including preparing the newborn’s body, was stressful for 11.4% of respondents, likely due to the direct contact with the deceased, which can be emotionally challenging.

A smaller proportion of respondents found nursing tasks (3.9%) and participating in baptism ceremonies (2.9%) stressful. These actions, though less commonly cited, carry emotional weight, especially in spiritual or ritual contexts.

Additionally, 4% mentioned other factors, such as various aspects of palliative care, involving multitasking and tailored approaches in complex clinical situations.

Overall, the findings indicate that the primary source of stress for medical staff is the emotional engagement with parents during their farewell. Technical and administrative duties, like preparing keepsakes and funeral documentation, also contribute to stress but to a lesser extent.

Coping Strategies for Stress in the Context of Caring for a Newborn with a Lethal Defect.

Table 6 presents the most commonly used methods of coping with stress employed by staff members in relation to their work, particularly in the context of caring for newborns with a lethal congenital defect. The results are expressed as percentages and refer to various emotional support methods and stress coping techniques. More than half of the respondents (52.8%) indicated that talking to loved ones and receiving support from close individuals is a key strategy for coping with stress. This is the most popular form of emotional support, highlighting the importance of interpersonal relationships in the process of emotional tension reduction.

The second most frequently indicated strategy is humour in the workplace. Almost one-third of the respondents (32.9%) use jokes as a way to ease difficult emotions, emphasising the role of distance and humour in coping with professional challenges.

Another significant strategy, used by 28.6% of respondents, is support from colleagues. A sense of community with other workers and their help in managing mental strain constitutes an important source of relief at work.

Entertainment and physical activity, such as sports, are popular methods of stress relief used by 25.7% and 23.4% of respondents, respectively. These activities can be viewed as forms of active relaxation and a way for individuals to detach themselves from professional issues.

For 22.1% of individuals, developing personal passions outside of work represents an effective stress coping strategy. This is an important way to unwind and relax, allowing for engagement in activities outside the work environment.

For 17.3% of people, faith provides support in coping with stress, which may reflect the spiritual dimension of dealing with difficult experiences. Additionally, relaxation techniques, used by 16% of respondents, may include meditation, breathing exercises, or other methods of tension reduction.

A less common, but still used strategy, is the use of spa treatments, as indicated by 11.7% of respondents. Such activities can support both physical and mental regeneration.

Less constructive ways of coping with stress, such as overeating (8.1%) and the use of substances like cigarettes or alcohol (7.8%), are chosen relatively infrequently, yet they remain present among some respondents.

The least commonly used strategy is seeking professional psychological help (2.3%). This result may suggest that these individuals rarely seek specialist support, possibly due to personal beliefs or barriers to accessing such help.

The findings show that medical staff most frequently rely on emotional support from loved ones and colleagues, as well as humour and recreational strategies. Fewer individuals turn to professional psychological help or engage in less constructive methods like overeating or substance use.

## 5. Discussion

The results of the study conducted on a group of 307 nurses and midwives provide valuable insights into the relationships between perceived stress levels and sociodemographic, professional, and coping strategies factors in the context of working with newborns with lethal congenital conditions. The primary aim of the study was to identify the coping strategies used by individuals working in this specific environment and to determine the impact of selected variables on perceived stress levels. The discussion of the results not only allows for the analysis of the obtained data in comparison with the existing literature but also highlights potential interventions aimed at reducing the psychological burden on medical staff.

The study revealed that stress levels were significantly dependent on the respondents’ education. Nurses and midwives with secondary education (obtained from a medical high school) more frequently reported high levels of stress (66.7%) compared to those with post-secondary education, among whom no cases of high stress were recorded. These findings can be interpreted as the result of differences in professional preparation, expectations, and attitudes toward work. Individuals with post-secondary education may adopt a more pragmatic approach to their professional duties, which reduces their susceptibility to stress. However, international literature shows some discrepancies—Chatzigiani and colleagues [29] demonstrated that nurse assistants, who undergo shorter professional training, experience higher stress levels. These differences may arise from diverse educational structures and specific approaches to work in different countries.

Work experience turned out to be another factor significantly influencing perceived stress levels. In this study, individuals with 2–5 years of professional experience more frequently reported medium levels of stress (47.7%), which may align with the adaptive curve model, where this period is characterised by the highest professional productivity but also by increased emotional burden. High stress levels were evenly distributed across all other experience categories, indicating that longer work experience may be associated with the development of adaptive mechanisms. According to Harris and Tao [30], nurses with shorter work experience (2–5 years) exhibit the highest levels of burnout, which correlates with the findings presented in this paper. In contrast, Chatzigiani and colleagues [29] argue that older nurses (over 50 years old) more frequently report high stress levels, which could result from the accumulation of difficult experiences and decreased psychological resilience.

Data analysis also showed that the workplace did not have a direct correlation with stress levels, suggesting that factors specific to the department, rather than the work environment itself, determine the psychological burden. The highest percentage of individuals with high stress levels worked in neonatal intensive care units (47.1%), which is consistent with the literature. McMeekin and colleagues [31] confirm that nurses working in these units experience higher stress levels compared to those in other specialties. In different cultural conditions, such as in Saudi Arabia, studies have shown moderate or low stress levels among nurses working in intensive care units [8], which may indicate differences in organisational structures and professional support across healthcare systems.

Motivational factors also turned out to be significant in the analysis of stress levels. Respondents who chose the profession for ideological reasons (“calling”) more often reported high levels of stress (55.0%). Such motivation may be linked to greater emotional involvement and the pressure of high moral standards. As Zbyrad [32] points out, idealists often dominate in service professions, which is confirmed by the findings of this study. In contrast, individuals driven by pragmatic motives showed lower stress levels, suggesting a more detached approach to professional duties.

Contact with newborns with lethal congenital conditions and their families was one of the most stress-inducing factors. The results indicate that the frequency of contact with patients who gave birth to a child with a lethal congenital condition had a significant impact on the stress levels of the participants. Nurses and midwives who encountered such cases several times a month or once a quarter more often reported high stress levels. This result aligns with the studies of Baranauskas and colleagues [33], who observed moderate to high stress levels among nurses after the death of a patient. Similar results were obtained in studies conducted in Greece [33] and Iran [2], where patient death was one of the main sources of stress in nurses’ work.

High stress levels were also correlated with experiencing psychological discomfort. Respondents reporting strong emotional discomfort made up as much as 74.4% of the group with high stress levels. This finding underscores the importance of psychological support when working with newborns with lethal congenital conditions. McAndrew and colleagues [34] found that intensive care nurses experience significant discomfort when professional tasks conflict with their ability to provide holistic care. Vincent and colleagues [35] highlighted the phenomenon of “moral distress”, which occurs when there is a conflict between knowledge of ethically correct behaviour and the procedures in place at a given workplace.

The study revealed that coping strategies had a significant impact on the professional functioning of nurses and midwives. The most commonly used strategies, such as active coping and planning, were adaptive and associated with lower stress levels. Avoidant strategies, such as denial, self-blame, or using psychoactive substances, were correlated with higher levels of psychological burden. These findings are consistent with research by Tesfaye [36] and Zhang and colleagues [37], who also emphasised the importance of active coping in stress reduction. An interesting strategy found was the empathetic approach to patients, which, according to Rivaldi and colleagues [38], helps reduce burnout levels.

Conversations with close ones (52.8%) and support from colleagues (28.6%) were the most commonly used coping methods in the study group. These results highlight the crucial role of interpersonal relationships in reducing psychological burdens. Similar conclusions were drawn by Andre and colleagues [39] and Laing and colleagues [40], who emphasised the importance of sharing professional experiences as a preventive measure against burnout.

The study results showed that the greatest stress in working with newborns with lethal congenital conditions comes from the need to accompany parents in their farewell to their child (69.1%). These experiences are not only emotionally burdensome but also require significant interpersonal skills. Research by Christoffersen et al. [41] indicates that an emotional approach to such situations, including “emotional release” (e.g., crying, journaling), can help in coping with difficulties. At the same time, a task-oriented approach, such as participating in farewell ceremonies with the family, also appears to be an essential element of psychological adaptation. Such an approach allows for closure of a difficult stage and reduces the risk of long-term emotional strain.

Another challenge in the work of neonatal staff was preparing keepsakes and providing palliative care for families. Christoffersen et al. [41] emphasise that confronting the emotions of parents when they lose their child is one of the most stressful aspects of working in neonatology. These studies align with the results of earlier authors who also pointed to the psychological burden associated with assisting families during the most difficult moments [42].

Patterson [43] in her research demonstrated that while traumatic experiences related to caring for dying patients often result in negative consequences, they can also contribute to personal and professional growth. According to the researcher, difficult experiences at work can lead to increased self-confidence, appreciation of life, and spiritual development. Similar conclusions were presented by authors of studies on stress in the nursing and midwifery profession in the context of their functioning after experiencing traumatic events [44]. Midwives involved in these studies reported increased inner strength, existential changes, and a sense of deeper meaning in life, which may have acted as an adaptive mechanism to working in challenging conditions.

In conclusion, the results of the study highlight the complexity of factors influencing stress levels among nurses and midwives working with newborns with lethal congenital conditions. They also emphasise the need for psychological and systemic interventions that could support medical staff in coping with professional challenges. Education on effective stress coping strategies, strengthening social support, and access to professional psychological help are key areas to consider when improving the well-being of this professional group.

An analysis of the study results in the context of contemporary health behaviour theories provides a better understanding of stress-coping mechanisms among nurses and midwives. One of the key concepts is the Coping with Stress Model developed by Endler and Parker, which distinguishes three coping styles: task-oriented, emotion-oriented, and avoidance-oriented. Research indicates that task-focused strategies, such as active problem-solving and planning, are more adaptive and effectively reduce occupational stress levels [45,46]. Furthermore, the Concept of Psychological Resilience highlights the role of individual resources, such as self-efficacy and flexibility in coping with stress. Strengthening these traits through training and support programmes can contribute to better management of professional demands [47,48].

Additionally, the Social Support Theory suggests that support from colleagues, family, and friends plays a crucial role in mitigating the effects of stress. Creating a work environment that encourages experience-sharing and emotional support can significantly improve the well-being of medical staff [49,50].

## 6. Practical Implications

The findings highlight the necessity of implementing psychological support programmes and stress management training for medical staff. It is particularly important to promote adaptive strategies, such as positive reappraisal and active coping, which can effectively reduce stress levels. Furthermore, healthcare institutions should consider introducing rotation systems to decrease the frequency of employees’ exposure to challenging clinical situations. The study underscores the need for further research to better understand the mechanisms of occupational stress and to develop effective support programmes.

Our study has certain limitations. It focuses on nurses and midwives providing care for newborns with lethal defects and their families exclusively in the Silesian agglomeration in Poland. As a result, its findings cannot be considered representative of the entire professional group of nurses working in perinatal palliative care across the country.

However, a key strength of this study is that its findings may serve as a foundation for many essential initiatives aimed at providing psychological and interventional support for nurses and midwives in perinatal palliative care in Poland, which is currently not institutionally available.

## 7. Conclusions

The study revealed that individuals with secondary education experienced higher levels of stress compared to those with higher education, possibly due to differences in professional preparation. Employees with 2–5 years of experience reported moderate stress, linked to the growing demands at this stage of their careers. Longer work experience did not significantly affect stress levels, suggesting the development of adaptive mechanisms. Neonatal intensive care units (NICUs) emerged as major stress factors, particularly in the care of critically ill newborns.

Motivation also played a significant role—those motivated ideologically experienced higher stress, while pragmatic approaches were associated with lower levels of stress. High stress levels were linked to emotional discomfort, and coping mechanisms such as talking to loved ones or receiving support from colleagues helped reduce stress. In the context of NICU work, interpersonal skills were crucial, especially when preparing keepsakes or providing palliative care. Therefore, it is important to provide appropriate emotional and psychological support to help employees effectively manage occupational stress.

## Figures and Tables

**Table 1:** Dependence of PSS 10 level on demographic variables.

Variables	Categories	Low (*n*, %)	Medium (*n*, %)	Hight (*n*, %)	χ^2^ Test Results df *p*	d Cohena
Education	Secondary: Medical High School	3 (7.7%)	10 (25.6%)	26 (66.7%)	30,651 10 0.001	0.2234
	Post-secondary vocational school	0 (0.0%)	10 (83.3%)	2 (16.7%)		
	Post-secondary vocational studies	7 (21.2%)	15 (45.5%)	11 (33.3%)		
	Bachelor’s degree	15 (18.8%)	20 (25.0%)	45 (56.3%)		
	Master’s degree	23 (16.3%)	61 (43.3%)	57 (40.4%)		
	Doctoral degree	0 (0.0%)	2 (100.0%)	0 (0.0%)		
Years of Service in the Current Department	Up to year	2 (18.2%)	7 (63.6%)	2 (18.2%)	24,023 14 0.046	0.1978
	2–5 years	13 (20.0%)	21 (32.3%)	31 (47.7%)		
	6–10 years	3 (7.0%)	23 (53.5%)	17 (39.5%)		
	11–15 years	7 (13.0%)	18 (33.3%)	29 (53.7%)		
	16–20 years	3 (7.3%)	17 (41.5%)	21 (51.2%)		
	21–25 years	9 (36.0%)	9 (36.0%)	7 (28.0%)		
	26–30 years	8 (19.5%)	12 (29.3%)	21 (51.2%)		
	Over 30 years	3 (11.1%)	11 (40.7%)	13 (48.1%)		
Workplace	Neonatology Department	15 (17.6%)	33 (38.8%)	37 (43.5%)	7984 8 0.435	0.1140
	Neonatal Intensive Care Unit	13 (10.7%)	51 (42.1%)	57 (47.1%)		
	Maternity Ward	9 (18.0%)	17 (34.0%)	24 (48.0%)		
	Delivery Block	7 (17.1%)	15 (36.6%)	19 (46.3%)		
	Perinatal Hospice	4 (40.0%)	2 (20.0%)	4 (40.0%)		
Reason for Choosing the Current Workplace	Job availability	26 (26.3%)	33 (33.3%)	40 (40.4%)	33,477 10 <0.001	0.2335
	Desire to help	4 (6.7%)	25 (41.7%)	31 (51.7%)		
	Calling	9 (11.3%)	27 (33.8%)	44 (55.0%)		
	Financial conditions	3 (9.1%)	18 (54.5%)	12 (36.4%)		
	Decision of superiors	0 (0.0%)	2 (16.7%)	10 (83.3%)		
	Other (distance, finances, working with children, chance, health)	6 (26.1%)	13 (56.5%)	4 (17.4%)		

**Table 2:** Dependence of PSS 10 level on professional variables.

Variables	Categories	Low *(n, %)*	Medium *(n, %)*	Hight *(n, %)*	χ^2^ Test Results df *p*	d Cohena
Frequency of Patients with a Prenatally Diagnosed Lethal Condition	Once a quarter	0 (0.0%)	16 (44.4%)	20 (55.6%)	35,123 6 <0.001	0.2392
	Several times a year	22 (14.0%)	78 (49.7%)	57 (36.3%)		
	Several times a month	26 (23.9%)	24 (22.0%)	59 (54.1%)		
	Several times a week	0 (0.0%)	0 (0.0%)	5 (100.0%)		
Frequency of Contact with Death and Dying of Neonates	Several times a year	35 (16.7%)	96 (45.7%)	79 (37.6%)	30,496 6 <0.001	0.2229
	Once a quarter	1 (2.9%)	14 (40.0%)	20 (57.1%)		
	Several times a month	12 (20.7%)	8 (13.8%)	38 (65.5%)		
	Several times a week	0 (0.0%)	0 (0.0%)	4 (100.0%)		
Frequency of Performing Nursing Tasks Related to the Care of a Newborn with a Lethal Condition	Several times a year	25 (13.6%)	86 (46.7%)	73 (39.7%)	31,510 6 <0.001	0.2265
	Once a quarter	2 (7.4%)	12 (44.4%)	13 (48.1%)		
	Several times a month	6 (10.9%)	15 (27.3%)	34 (61.8%)		
	Several times a week	15 (36.6%)	5 (12.2%)	21 (51.2%)		
Degree of Discomfort Experienced in Care of a Newborn with a Lethal Condition	None	6 (21.4%)	8 (28.6%)	14 (50.0%)	30,041 8 <0.001	0.2212
	Small/Minimal	6 (13.0%)	13 (28.3%)	27 (58.7%)		
	Medium	24 (21.6%)	49 (44.1%)	38 (34.2%)		
	Large	8 (9.6%)	42 (50.6%)	33 (39.8%)		
	Very large	4 (10.3%)	6 (15.4%)	29 (74.4%)		
Factors Affecting Psychological Burden Considered to Cause Stress in the Care of a Newborn with a Lethal Condition	Time pressure	0 (0.0%)	7 (30.4%)	16 (69.6%)	7326 2 0.026	0.1092
	Close contact with death	1 (4.8%)	16 (76.2%)	4 (19.0%)	13,610 2 0.001	0.1489
	Helplessness or limited ability to help	20 (24.4%)	26 (31.7%)	36 (43.9%)	6903 2 0.032	0.1060
	Excessive emotional involvement	2 (7.1%)	9 (32.1%)	17 (60.7%)	3205 2 0.201	0.0722
	Close contact with parents’ suffering	24 (17.6%)	62 (36.3%)	85 (49.7%)	2309 2 0.315	0.0613
	Close contact with the child’s suffering	16 (11.1%)	63 (43.8%)	65 (45.1%)	5579 2 0.061	0.0953

**Table 3:** Basic descriptive statistics if stress MiniCope coping strategies.

	*N*	*M*	*SD*	*Me*	Min.	Max	Skewness	Kurtosis
MiniCope Active Coping	307	2.06	0.635	2.00	0.00	3.00	−0.91	1.53
MiniCope Planning	307	1.95	0.590	2.00	0.00	3.00	−0.64	1.54
MiniCope Positive Reappraisal	307	1.81	0.690	2.00	0.00	3.00	−0.54	0.26
MiniCope Acceptance	307	1.86	0.574	2.00	0.00	3.00	−0.34	0.74
MiniCope Sense of Humour	307	0.88	0.681	1.00	0.00	2.50	0.51	−0.56
MiniCope Turning to Religion	307	1.12	0.915	1.00	0.00	3.00	0.54	−0.61
MiniCope Seeking Emotional Support	307	1.79	0.716	2.00	0.00	3.00	−0.41	−0.09
MiniCope Seeking Instrumental Support	307	1.76	0.663	2.00	0.00	3.00	−0.18	−0.17
MiniCope Occupying	307	1.76	0.683	2.00	0.00	3.00	−0.34	0.16
MiniCope Denial	307	0.88	0.751	1.00	0.00	3.00	0.50	−0.53
MiniCope Venting	307	1.49	0.620	1.50	0.00	3.00	−0.29	0.02
MiniCope Substance Use Psychoactive	307	0.66	0.712	0.50	0.00	2.50	0.69	−0.56
MiniCope Desistance	307	0.95	0.689	1.00	0.00	3.00	0.42	−0.33
MiniCope Self-Blame	307	1.20	0.714	1.00	0.00	3.00	0.34	−0.26

**Table 4:** Correlations between PSS10 score and MiniCope coping strategies.

Correlations			
			PSS10
Spearman’s rho	MiniCope Active Coping	Correlation coefficient	−0.098
		Significance (bilateral)	0.087
	MiniCope Planning	Correlation coefficient	−0.046
		Significance (bilateral)	0.421
	MiniCope Positive Reappraisal	Correlation coefficient	−0.211
		Significance (bilateral)	0
	MiniCope Acceptance	Correlation coefficient	−0.055
		Significance (bilateral)	0.335
	MiniCope Sense of Humour	Correlation coefficient	0.065
		Significance (bilateral)	0.258
	MiniCope Turning to Religion	Correlation coefficient	0.15
		Significance (bilateral)	0.008
	MiniCope Seeking Emotional Support	Correlation coefficient	−0.129
		Significance (bilateral)	0.024
	MiniCope Seeking Instrumental Support	Correlation coefficient	0.061
		Significance (bilateral)	0.285
	MiniCope Occupying	Correlation coefficient	0.021
		Significance (bilateral)	0.713
	MiniCope Denial	Correlation coefficient	0.215
		Significance (bilateral)	0
	MiniCope Venting	Correlation coefficient	0.153
		Significance (bilateral)	0.007
	MiniCope Substance Use Psy-choactive	Correlation coefficient	0.122
		Significance (bilateral)	0.032
	MiniCope Desistance	Correlation coefficient	0.266
		Significance (bilateral)	0
	MiniCope Self-Blame	Correlation coefficient	0.32
		Significance (bilateral)	0

**Table 5:** Activities during farewell with the child.

Activity	Percentage (%)
Accompanying parents during their farewell with the child	69.1
Preparing keepsakes for parents (e.g., footprints)	50
Providing care free of disproportionate life-prolonging treatment	40
Providing documentation for funeral arrangements	30
Performing post-mortem care	20
Carrying out nursing activities	15
Participation in baptism ceremonies	10
Other (e.g., individualised care, all mentioned to varying extents)	5

**Table 6:** Coping mechanism in stressful situations.

Coping Mechanism in Stressful Situations	Percentage (%)
Talking with loved ones/support from close people	52.8
Joking at work	32.9
Support from colleagues	28.6
Entertainment	25.7
Sport	23.4
Hobbies	22.1
Faith	17.3
Relaxation techniques	16
SPA treatments	11.7
Overeating	8.1
Using stimulants (cigarettes, alcohol)	7.8
Psychologist’s help	2.3

## Data Availability

The data generated during and analysed during the current study are available from the corresponding author on reasonable request.

## Public Involvement Statement

No public involvement in any aspect of this research.

## Guidelines and Standards Statement

All methods were carried out in accordance with relevant guidelines and regulations.
