# Correction: Urbańska et al. Influence of Sociodemographic Factors on Level Stress and Coping Strategies of Nurses and Midwives Caring for Newborns with Lethal Defects. *Nurs. Rep.* 2025, *15*, 116

**DOI:** 10.3390/nursrep15050170

**Published:** 2025-05-13

**Authors:** Katarzyna Anna Urbańska, Beata Naworska, Agnieszka Drosdzol-Cop

**Affiliations:** 1 Neonatology Unit, BCM The Guardian Angels Hospital of the Brothers Hospitallers of St. John of God in Katowice, Markiefki 87, 40-211 Katowice, Poland; 2 Department of Gynaecology and Obstetrics, Faculty of Health Sciences in Katowice, Medical University of Silesia in Katowice, 40-751 Katowice, Poland; bnaworska@sum.edu.pl; 3 Chair and Department of Gynecology, Obstetrics and Oncological Gynecology, Faculty of Medical Sciences in Katowice, Medical University of Silesia in Katowice, 40-211 Katowice, Poland; adrosdzol@sum.edu.pl

There was an error in the original publication [1]. The version posted on the Nursing Reports website contains the same description of the results in Table 5 and Table 6.

A correction has been made to Section 4, Paragraph 67–70 (Description of Table 6).

Table 6 presents the most commonly used methods of coping with stress employed by staff members in relation to their work, particularly in the context of caring for newborns with a lethal congenital defect. The results are expressed as percentages and refer to various emotional support methods and stress coping techniques. More than half of the respondents (52.8%) indicated that talking to loved ones and receiving support from close individuals is a key strategy for coping with stress. This is the most popular form of emotional support, highlighting the importance of interpersonal relationships in the process of emotional tension reduction.

The second most frequently indicated strategy is humour in the workplace. Almost one-third of the respondents (32.9%) use jokes as a way to ease difficult emotions, emphasising the role of distance and humour in coping with professional challenges.

Another significant strategy, used by 28.6% of respondents, is support from colleagues. A sense of community with other workers and their help in managing mental strain constitutes an important source of relief at work.

Entertainment and physical activity, such as sports, are popular methods of stress relief used by 25.7% and 23.4% of respondents, respectively. These activities can be viewed as forms of active relaxation and a way for individuals to detach themselves from professional issues.

For 22.1% of individuals, developing personal passions outside of work represents an effective stress coping strategy. This is an important way to unwind and relax, allowing for engagement in activities outside the work environment.

For 17.3% of people, faith provides support in coping with stress, which may reflect the spiritual dimension of dealing with difficult experiences. Additionally, relaxation techniques, used by 16% of respondents, may include meditation, breathing exercises, or other methods of tension reduction.

A less common, but still used strategy, is the use of spa treatments, as indicated by 11.7% of respondents. Such activities can support both physical and mental regeneration.

Less constructive ways of coping with stress, such as overeating (8.1%) and the use of substances like cigarettes or alcohol (7.8%), are chosen relatively infrequently, yet they remain present among some respondents.

The least commonly used strategy is seeking professional psychological help (2.3%). This result may suggest that these individuals rarely seek specialist support, possibly due to personal beliefs or barriers to accessing such help.

The findings show that medical staff most frequently rely on emotional support from loved ones and colleagues, as well as humour and recreational strategies. Fewer individuals turn to professional psychological help or engage in less constructive methods like overeating or substance use.

The authors state that the scientific conclusions are unaffected. This correction was approved by the Academic Editor. The original publication has also been updated.
